# What Matters Most to Informal Caregivers of Persons with Dementia? Results from the Application of Deep Learning to Twitter Posts from 2013 to 2022

**DOI:** 10.1002/alz.083965

**Published:** 2025-01-09

**Authors:** Li Chang Ang, Rahul Malhotra, Anupama Roy Chowdhury, Tau Ming Liew

**Affiliations:** ^1^ Singapore General Hospital, Singapore, Singapore Singapore; ^2^ Duke‐NUS Medical School, Singapore, Singapore Singapore

## Abstract

**Background:**

With the advent of new media, more people – possibly including caregivers of persons with dementia – are turning to social media platforms to share their thoughts and emotions related to personal life experiences. This may potentially serve as an opportunity to leverage on social media to gain insights into the key issues faced by dementia caregivers. We examined salient concerns of dementia caregivers through Twitter posts, aiming to shed light on how to better support and engage such caregivers.

**Method:**

English tweets related to “dementia” and “caregiver” (or related terms such as “Alzheimer’s disease” and “carer”) were extracted between 1^st^ January 2013 and 31^st^ December 2022. A supervised deep learning model (Bidirectional Encoder Representations from Transformers, BERT) was trained to select tweets describing individual’s accounts related to dementia caregiving. Then, an unsupervised deep learning approach (BERT‐based topic modelling) was applied to identify topics from selected tweets, with each topic further grouped into themes manually using thematic analysis.

**Result:**

A total of 44,527 tweets were analysed, and stratified using the emergence of COVID‐19 pandemic as a threshold. Three themes were derived: challenges of caregiving in dementia, positive aspects related to dementia caregiving, and dementia‐related stigmatization. Over time, there is a rising trend of tweets relating to dementia caregiving. Post‐pandemic, challenges of caregiving remained the top discussed topic; followed by an increase in tweets related to dementia‐related stigmatization; and a decrease in tweets related to positive aspects of caregiving (p‐value<.001).

**Conclusion:**

Social media is increasingly being used by dementia caregivers to share their thoughts. The findings uncover the worrying trend of growing dementia‐related stigmatization among the caregivers, and its manifestation in the form of devaluing others. The persistence of these issues post‐pandemic underscores the need for caregiver’s support and resources.

